# Silica-coated upconversion lanthanide nanoparticles: The effect of crystal design on morphology, structure and optical properties

**DOI:** 10.3762/bjnano.6.235

**Published:** 2015-12-03

**Authors:** Uliana Kostiv, Miroslav Šlouf, Hana Macková, Alexander Zhigunov, Hana Engstová, Katarína Smolková, Petr Ježek, Daniel Horák

**Affiliations:** 1 Institute of Macromolecular Chemistry, Academy of Sciences of the Czech Republic, Heyrovského nám. 2, 162 06 Prague 6, Czech Republic; 2 Institute of Physiology, Academy of Sciences of the Czech Republic, Vídeňská 1083, 142 20 Prague 4, Czech Republic

**Keywords:** lanthanide, nanoparticles, oleylamine, silica, upconversion

## Abstract

NaYF_4_:Yb^3+^/Er^3+^ nanoparticles were synthesized by thermal decomposition of lanthanide trifluoroacetates using oleylamine (OM) as both solvent and surface binding ligand. The effect of reaction temperature and time on the properties of the particles was investigated. The nanoparticles were characterized by transmission electron microscopy (TEM), electron diffraction (ED), energy dispersive spectroscopy (EDX), dynamic light scattering (DLS), thermogravimetric analysis (TGA), elemental analysis and X-ray diffraction (XRD) to determine morphology, size, polydispersity, crystal structure and elemental composition of the nanocrystals. TEM microscopy revealed that the morphology of the nanoparticles could be fine-tuned by modifying of the synthetic conditions. A cubic-to-hexagonal phase transition of the NaYF_4_:Yb^3+^/Er^3+^ nanoparticles at temperatures above 300 °C was confirmed by both ED and XRD. Upconversion luminescence under excitation at 980 nm was observed in the luminescence spectra of OM–NaYF_4_:Yb^3+^/Er^3+^ nanoparticles. Finally, the OM–NaYF_4_:Yb^3+^/Er^3+^ nanoparticles were coated with a silica shell to enable further functionalization and increase biocompatibility and stability in aqueous media, preventing particle aggregation.

## Introduction

Due to their unique physicochemical properties, nanometer-scale materials are finding widespread applications as drug delivery systems in the diagnosis and treatment of various diseases [1–2]. Recently, upconversion nanoparticles have shown promise as optical materials [3] and a number of reviews [4–6] have described their applications in drug and gene delivery [7], cell labeling and tracking [8], bioimaging [9] and photodynamic therapy [10]. Lanthanide-doped upconversion nanoparticles emit visible light upon excitation by near-IR light (NIR). Compared with organic dyes and semiconductor quantum dots, upconversion nanoparticles have attractive chemical and optical properties, as well as low toxicity [11], sharp emission bandwidths, large anti-Stokes shifts [12] and high resistance to photobleaching and photoblinking [13]. NIR light can non-invasively penetrate living organisms deeply because the excitation wavelength is within the optical transparency window of tissues (700–1000 nm) [14]. Upconversion proceeds by different mechanisms, such as energy transfer and excited-state absorption and photon avalanche. These three mechanisms are based on the sequential absorption of two or more photons. Upconversion emission proceeds by sequential absorption of two photons and leads to population of a highly excited state. If the activator sequentially obtains energy from the sensitizer, it reaches the excited state and emits light at a short wavelength [15]. In the NaYF_4_-based particles, the Yb^3+^ sensitizer is often used to absorb photons at 980 nm, providing an excited state from which energy is transferred to a neighboring activator (e.g., Er^3+^). Hexagonal β-NaYF_4_ crystals sensitized by Yb^3+^ and activated by Er^3+^/Tm^3+^ ions are a highly efficient host material for green and blue upconversion [16–18].

There are many techniques to synthesize small-sized high-quality upconversion particles. The most widely used of these, wet-chemical methods, involve thermal decomposition of rare earth organic acid precursors, typically metal trifluoroacetates, acetylacetonates, oleates or acetates, in non-aqueous media in the presence of surfactants and at high temperatures [19]. In addition to their effect on reaction temperature and time, the advantages of trifluoroacetates include the rapid formation of reactive fluoride compounds and the ability to control morphology, shape, crystal phase and size depending on the ratios of the starting reagents (i.e., organic precursors, surfactants and solvents) [20]. The surfactants, e.g., oleylamine (OM) and oleic acid, typically consist of polar capping groups and long hydrocarbon chains. Other synthetic methods include the hydro(solvo)thermal technique, which involves mixing lanthanide and fluoride salts (e.g., NH_4_F) in a high-boiling point solvent (e.g., ethylene glycol) at high temperature and pressure [21–22].

Upconversion particles are typically prepared in different morphologies, sizes and shapes with high surface areas to efficiently conjugate target ligands and drugs [23]. It is important to tailor the surface of the nanoparticles to the specific application. Thus, various ligands and functionalities have to be attached to the particle surface to provide efficient drug delivery, to ensure engulfment by the cells, or to control the release of biomolecules and their specific target. Finally, the surface modification must ensure that the particles can be dispersed in the aqueous biological media. Charged or polar moieties, such as amphiphilic (co)polymers [24], lipids [25] and silica [26], are therefore attached to the particle surface. The silica coating imparts many useful properties to the nanoparticles, including additional functionalization and biocompatibility [27].

We herein report the preparation of OM–NaYF_4_:Yb^3+^/Er^3+^ nanoparticles with controlled morphology, size, composition, phase and luminescence. Thorough particle characterization was performed to elucidate the relationship between synthetic conditions and particle structure. Surface modification of the particles using silica precursors enhanced colloidal stability and facilitated the transfer of the crystals in aqueous media.

## Experimental

### Materials

Sodium and yttrium(III) trifluoroacetate, ytterbium(III) chloride hexahydrate, erbium(III) chloride hexahydrate, oleylamine (OM), tetramethoxysilicate (TMOS; > 99%) and Igepal CO-520 (polyoxyethylene(5) nonylphenyl ether) were purchased from Sigma-Aldrich (St. Louis, MO, USA). Ytterbium(III) oxide was prepared by dissolving ytterbium(III) chloride hexahydrate (1 g) in water (10 mL) followed by the addition of 25% NH_4_OH (1.2 mL). Erbium(III) oxide was prepared analogously from the respective chloride (0.5 g) dissolved in water (5 mL), which was followed by the addition of 25% NH_4_OH (0.6 mL). The resulting ytterbium(III) oxide was washed with water to remove ammonium chloride, and trifluoroacetic acid (0.58 ml) was added to reach pH 1. Water was removed on a rotary evaporator, and the resulting ytterbium(III) trifluoroacetate was dried in a desiccator for 24 h. Erbium(III) trifluoroacetate was obtained similarly with the only difference being the addition of 0.29 mL of trifluoroacetic acid. All other chemicals employed in this study were obtained from Lachema (Brno, Czech Republic). Ultrapure Q-water ultrafiltered on a Milli-Q Gradient A10 system (Millipore, Molsheim, France) was employed in all experiments.

### Synthesis of OM-stabilized upconversion nanoparticles

Synthesis of the upconversion nanoparticles was similar to a previously reported protocol [20] with slight modifications. To prepare the hexagonal NaYF_4_:20%Yb/2%Er particles, a mixture of CF_3_COONa (2 mmol), (CF_3_COO)_3_Y (0.78 mmol), (CF_3_COO)_3_Yb (0.2 mmol) and (CF_3_COO)_3_Er (0.02 mmol) was dissolved in OM (10 mL) in a 100 mL three-neck round-bottom flask and heated to 120 °C under vigorous magnetic stirring to remove water. Heating then continued at a predetermined temperature (e.g., 330 °C) for a given amount of time (0.5–4 h) under argon to prevent oxidation. The resulting transparent yellowish reaction mixture was cooled and ethanol (20 mL) was added. The OM–NaYF_4_:Yb^3+^/Er^3+^ nanoparticles were separated by centrifugation, washed three times with hexane and deionized water and transferred in hexane.

### Synthesis of silica-coated upconversion nanoparticles

The surfaces of the OM–NaYF_4_:Yb^3+^/Er^3+^ nanoparticles were coated with silica using a reverse microemulsion method [28] with slight modifications. The OM–NaYF_4_:Yb^3+^/Er^3+^ nanoparticles (50 mg) were dispersed in cyclohexane (10 mL). Igepal CO-520 (0.5 mL) and 25% aqueous ammonia (0.08 mL) were added, and the suspension was mixed using a Sonopuls sonicator (Bandelin, Berlin, Germany) for 30 min, yielding a stable colloid. TMOS (0.04 mL) was then added, and the mixture was stirred (600 rpm) at room temperature for 2 days. The resulting NaYF_4_:Yb^3+^/Er^3+^&SiO_2_ nanoparticles were precipitated by the addition of acetone (10 mL), separated by centrifugation and washed five times with ethanol and ethanol/water (1:1 v/v) to remove the surfactant.

### Characterization of the nanoparticles

The nanoparticles were visualized and analyzed on a Tecnai G^2^ Spirit Twin transmission electron microscope (TEM; FEI; Brno, Czech Republic) equipped with an energy dispersive spectrometer (EDX; Mahwah, NJ, USA). Bright field TEM imaging (BF), electron diffraction (ED) and energy dispersive spectroscopy (EDX) were used to determine the morphology, crystal structure and elemental composition of the nanocrystals, respectively. All TEM micrographs, diffractograms and spectra were taken at an accelerating voltage of 120 kV. Particle size distribution was analyzed with the Atlas software (Tescan Digital Microscopy; Brno, Czech Republic). The number-average diameter (*D*_n_) and weight-average diameter (*D*_w_) and uniformity (polydispersity index PDI = *D*_w_/*D*_n_) were calculated from at least 500 individual particles. *D*_n_ and *D*_w_ can be expressed as follows:

[1]
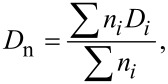


[2]
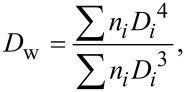


where *n_i_* and *D_i_* are the number and diameter of the particles, respectively. The ED patterns were processed with ProcessDiffraction software [29] and compared with the diffraction patterns of known NaYF_4_ crystal structures calculated with PowderCell software [30] or downloaded from crystallographic databases.

The hydrodynamic particle size (*D*_h_) was measured using dynamic light scattering (DLS) on a ZEN 3600 Zetasizer Nano Instrument (Malvern Instruments; Malvern, UK) at pH 1. Thermogravimetric analysis (TGA) was performed in air using a Perkin Elmer TGA 7 analyzer (Norwalk, CT, USA) from 30 to 850 °C at a heating rate of 10 °C·min^−1^. Elemental analysis was determined on a Perkin-Elmer 2400 CHN apparatus.

X-ray diffraction (XRD) was measured on a Rigaku MolMet (Molecular Metrology System) instrument (Tokyo, Japan) using a pinhole camera attached to a microfocused Osmic MicroMax 002 X-ray beam generator operating at 45 kV and 0.66 mA (30 W). The camera was equipped with a removable imaging plate 23 × 25 cm (Fujifilm). The experimental setup covered a momentum transfer (*q*) range of 0.25–3.5 Å^−1^, where *q* = (4π/λ)sinθ, wavelength λ = 1.54 Å and 2θ was the scattering angle. The center and sample-to-detector distance were calibrated using Si powder. The samples were measured in a transmission mode. Crystallinities (*C*_r_) were estimated using integral intensities (sum of areas) diffracted by crystalline (*I*_c_) and amorphous (*I*_a_) phases according to Equation 3:

[3]
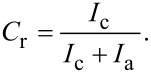


The crystal size was calculated [31] according to the Scherrer Equation 4:

[4]
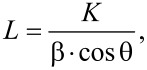


where *K* is the shape factor (typically 0.9), β is the full width of half maximum (FWHM) of reflection (in radians) and λ is the wavelength of the X-rays (1.54 Å).

Infrared spectra were obtained on a Nexus Nicolet 870 FTIR spectrometer (Thermo Fisher Scientific; Waltham, MA, USA) equipped with a liquid nitrogen-cooled mercury cadmium telluride (MCT) detector using a Golden Gate single reflection ATR cell (Specac; Slough, UK). Spectra (256 scans) were acquired at room temperature at a resolution of 4 cm^−1^. Water vapor (atmospheric spectrum) and background were subtracted from the spectra, and an ATR correction was applied.

Upconversion luminescence spectra were recorded on a Leica TCS SP2 AOBS confocal inverted fluorescent microscope (Leica Microsystems; Mannheim, Germany) using a PL APO 100×/1.40–0.70 oil immersion objective (a pinhole 1 Airy unit) and a Chameleon laser Ultra Ti:sapphire laser (Coherent; Santa Clara, CA, USA) at 980 nm excitation. The nanoparticle (0.01 g) dispersion in hexane was placed on a cover glass using a spin coating technique.

## Results and Discussion

To prepare NaYF_4_:Yb^3+^/Er^3+^ nanoparticles with a hexagonal unit cell, lanthanide trifluoroacetates were decomposed in OM, serving as both solvent and surface binding ligand [20]. Because the particle size plays a key role in biomedical applications, e.g., for internalization of nanoparticles by cells of the reticuloendothelial system [32], the effects of both reaction temperature and time were investigated to control morphology, particle size and crystallinity.

### Effect of reaction temperature

**Figure 1:**
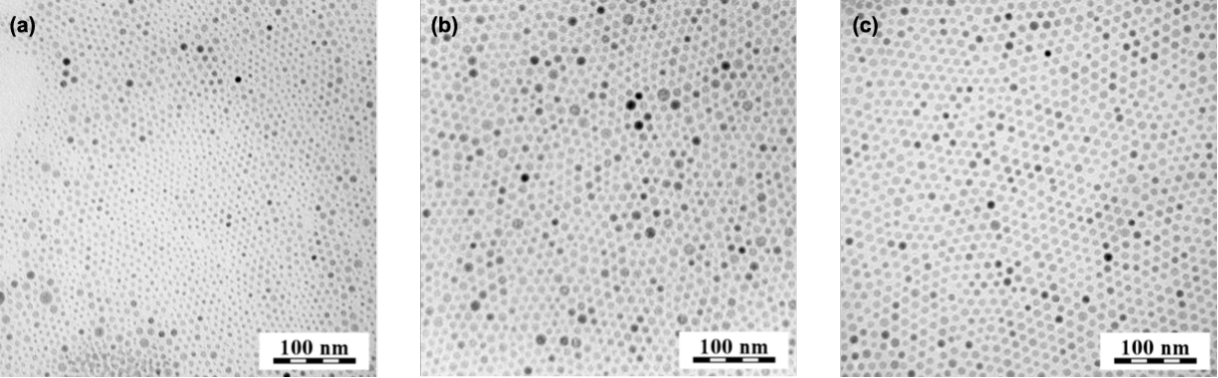
TEM micrographs of OM-NaYF_4_:Yb^3+^/Er^3+^ nanoparticles prepared at (a) 250, (b) 300 and (c) 350 °C for 1 h.

**Figure 2:**
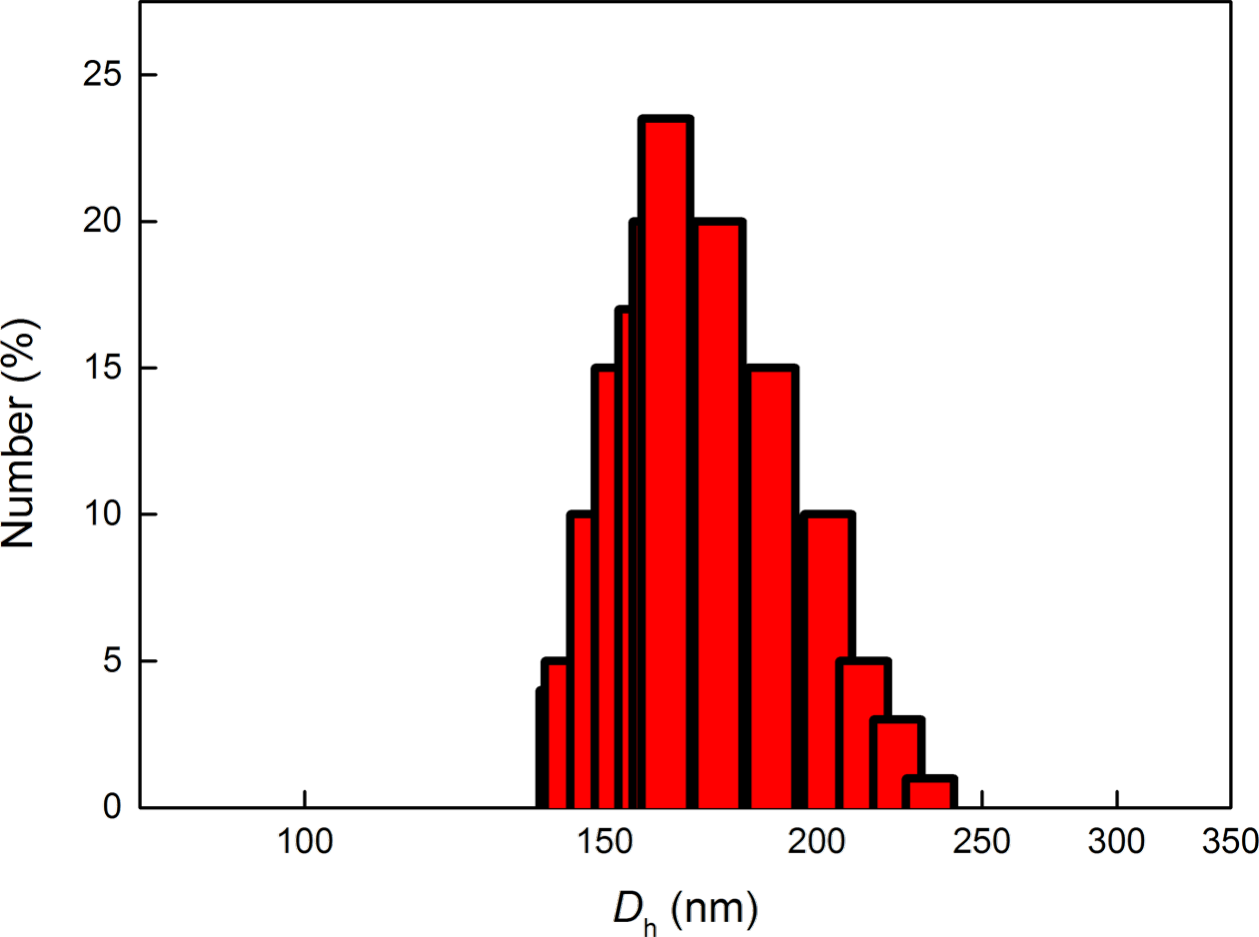
Particle size distribution of no. 4 OM-NaYF_4_:Yb^3+^/Er^3+^ nanoparticles (Table 1) determined by DLS.

In the first series of experiments, reaction temperature was varied from 250 to 350 °C, while the other reaction parameters, such as the composition NaYF_4_:20%Yb^3+^/2%Er^3+^, the concentration of sodium and lanthanide trifluoroacetates in OM (8.8 wt %) and the reaction time (1 h), were held constant. The selected composition of rare-earth ions produced highly efficient IR-to-visible upconversion [20]. As illustrated in the TEM/BF micrographs (Figure 1), the particles were well separated, suggesting that the long OM chains on the particle surface effectively prevented aggregation. The particles had regular spherical shapes with sizes (*D*_n_) in the range of 6–10 nm (Table 1). The particle size increased as the temperature increased up to 350 °C, which is close to the OM boiling point. Particle size distribution was relatively narrow, as documented by the polydispersity index (PDI) ranging from 1.07 to 1.30. Nearly monodispersed particles (PDI = 1.07), which are supposed to possess uniform physical, chemical and biological properties, were obtained at 350 °C. Small size (about 10 nm) and narrow size distribution are important for particles to be considered as probes of target proteins, oligonucleotides and other biomolecules in cells and tissues. DLS experiments showed that the average hydrodynamic particle size in water was large (*D*_h_ = 163–265 nm), suggesting the formation of particle aggregates. DLS provides the z-average of the particle size, which is substantially larger than the number-average diameter determined by TEM. For example, the hydrodynamic diameter of the no. 4 OM-NaYF_4_:Yb^3+^/Er^3+^ nanoparticles (Table 1) was 135–240 nm, with the most frequent fraction between 150–200 nm (Figure 2). The large difference between the number-average diameter (TEM) and the hydrodynamic diameter (DLS) is due to the fact that hydrophobic particles tend to aggregate and cannot be resolved as individual particles by DLS.

**Table 1:** Effect of reaction temperature on OM–NaYF_4_:Yb^3+^/Er^3+^ nanoparticles prepared for 1 h.

no.	*T* (°C)	*D*_n_ (nm)	PDI	*D*_h_ (nm)	PI	*L*(cubic) (nm)	*L*(hex) (nm)	crystallinity (wt %)	C (wt %)	N (wt %)	coating (wt %)			*I*_545_/*I*_660_
											C	N	TGA	

1	250	6	1.28	237	0.43	8.8	12.1	76	3.87	0.24	4.8	4.6	8.7	1.93
2	280	8	1.21	265	0.43	9.7	14.0	77	2.60	0.13	3.2	2.5	5.2	2.50
3	300	9	1.16	199	0.35	6.4	17.2	67	4.20	0.30	5.2	5.9	11.1	1.83
4	330	10	1.30	188	0.27	6.4	18.6	74	3.00	0.15	3.7	2.9	7.1	1.79
5	350	9	1.07	163	0.24	–	21.3	71	18.89	2.42	23.4	21	35.2	1.68

*D*_n_: number-average diameter (TEM); PDI: polydispersity index (TEM); *D*_h_: hydrodynamic diameter (DLS); PI: polydispersity (DLS); *L*: average crystallite size (XRD); C: carbon content; N: nitrogen content; TGA: thermogravimetric analysis; *I*_545_/*I*_660_: characteristic upconversion ratio.

**Figure 3:**
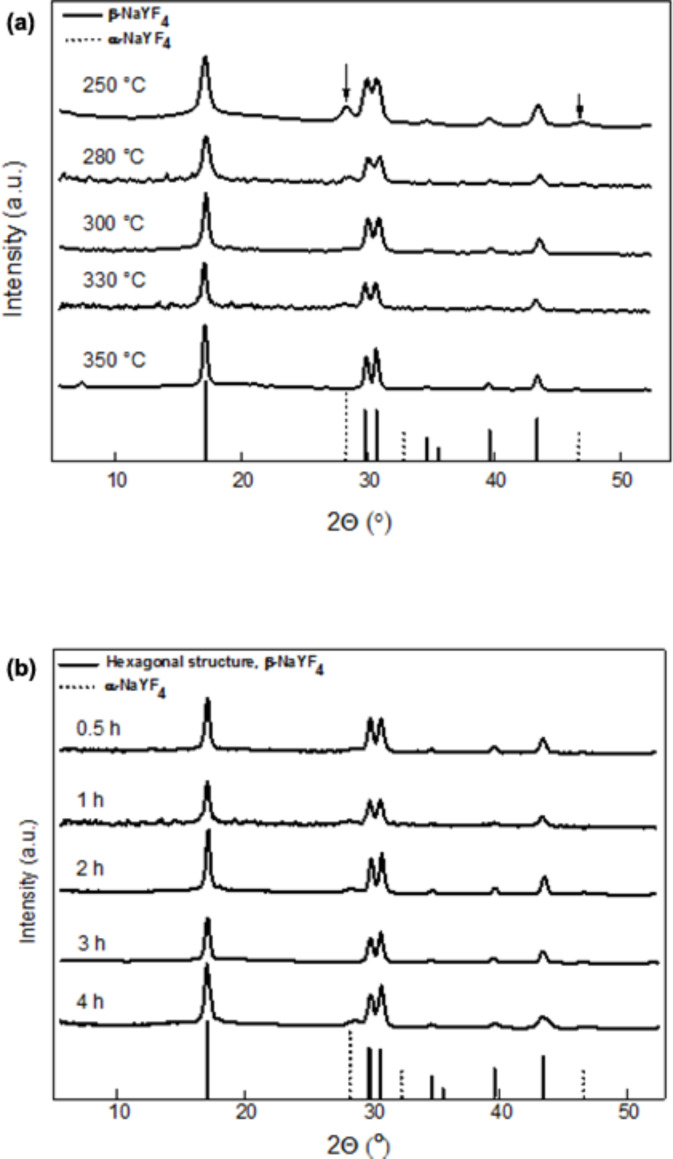
X-ray diffraction patterns of OM-NaYF_4_:Yb^3+^/Er^3+^: effect of (a) reaction temperature (time 1 h) and (b) time (temperature 330 °C). Lines corresponding to hexagonal and cubic phases were found in [36]. The arrows at the top of the image denote the most intensive diffractions of the cubic phase.

Morphology and crystalline structure of the dry OM–NaYF_4_:Yb^3+^/Er^3+^ nanocrystals were determined by TEM/BF, TEM/ED and XRD. The crystal structure of NaYF_4_ typically exhibits two polymorphic forms: the metastable cubic α-phase and the thermodynamically stable hexagonal β-phase. The latter is a much better host lattice for the luminescence of various optically active lanthanide ions. Altering reaction temperature is a successful approach to control crystal structures. Higher reaction temperature and longer reaction time are required to provide sufficient free energy to overcome the activation barrier of an α/ß phase transition [19,33]. In the XRD experiments, all crystal diffractions and amorphous halos of the OM–NaYF_4_:Yb^3+^/Er^3+^ nanoparticles were fitted by Voigt [34] and Gaussian peak functions, respectively, yielding the position, full width of half maximum (FWHM), and area for each peak. The effect of reaction temperature on OM–NaYF_4_:Yb^3+^/Er^3+^ crystallinity is shown in Figure 3a. The degree of crystallinity of the particles reached 67–77 wt % (Table 1). The amorphous halo originated from OM surrounding the NaYF_4_:Yb^3+^/Er^3+^ crystallites. Sizes of the cubic and hexagonal phases were estimated using the Scherrer equation from the [111] (2θ = 28.2) and [101] (2θ = 30.9) reflections, respectively [35]. XRD diffractograms of the OM–NaYF_4_:Yb^3+^/Er^3+^ nanoparticles prepared at lower temperatures exhibited both α- and β-phases, but the average α-phase crystallite size decreased from about 9 to 6 nm with increasing temperature (Table 1). In contrast, the average size of the β-phase crystals increased from 12 to 21 nm, which was in rough agreement with the *D*_n_ determined by TEM. As seen in the diffractograms, nanoparticles with both cubic and hexagonal phases were obtained at 250 and 280 °C, respectively. When the particles were synthesized at temperatures above 300 °C, the intensity of the peaks corresponding to the hexagonal OM–NaYF_4_:Yb^3+^/Er^3+^ phase increased significantly. The particles obtained at 330 and 350 °C exhibited the hexagonal phase, but traces of the cubic phase were still present in the lower temperature particles. The position of the XRD peaks (Figure 3a) corresponded to the hexagonal phase, as published in literature [20].

**Figure 4:**
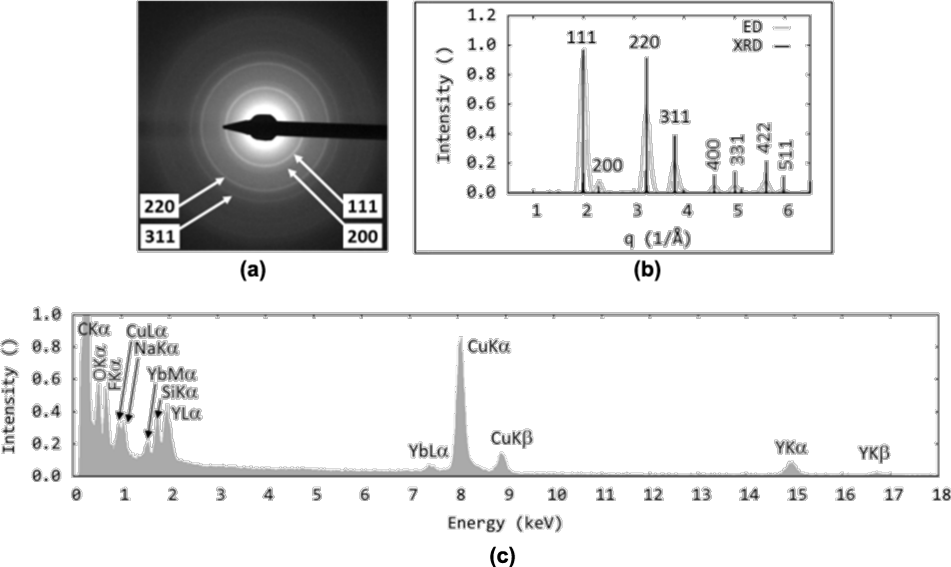
TEM analysis: (a) ED pattern, (b) comparison of experimental ED pattern and calculated XRD pattern, and (c) EDX spectrum of no. 1 OM–NaYF_4_:Yb^3+^/Er^3+^ nanoparticles. Peaks were indexed according to [20].

**Figure 5:**
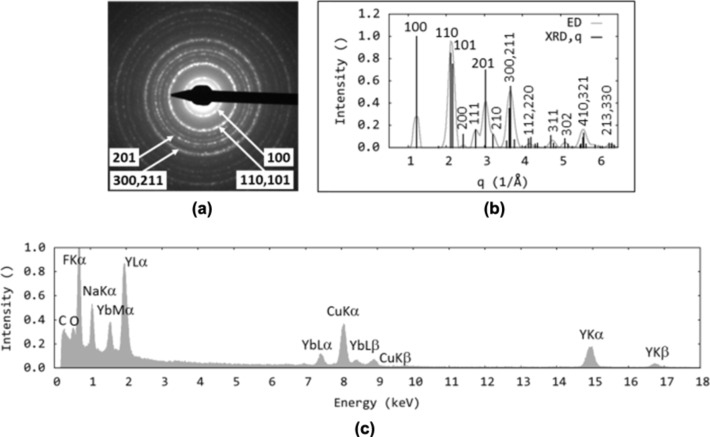
TEM analysis: (a) ED pattern, (b) comparison of experimental ED pattern and calculated XRD pattern, and (c) EDX spectrum of OM-NaYF_4_:Yb^3+^/Er^3+^ nanoparticles no. 4. Peaks were indexed according to [20].

The results presented above are in good agreement with those from TEM/ED (Figure 4a,b and Figure 5a,b). Both methods illustrated the significant impact of the reaction temperature on the properties of the particles. The particles prepared at low temperatures (250 °C) exhibited cubic crystal structures [19,33] (Figure 4a,b), whereas the particle structure obtained at higher temperatures (≥ 330 °C) was hexagonal and corresponded to Na(Y_0.57_Yb_0.39_Er_0.04_)F_4_ (Figure 5a,b). The EDX spectra contained strong peaks for the main components (Na, Y, F) and detectable Yb, but the amount of Er was below the detection limit (Figure 4c and Figure 5c). The spectra also contained signals from the sample holder (Cu), supporting carbon film (C), small amounts of oleylamine (C, O, N) and inorganic impurities (Si).

**Figure 6:**
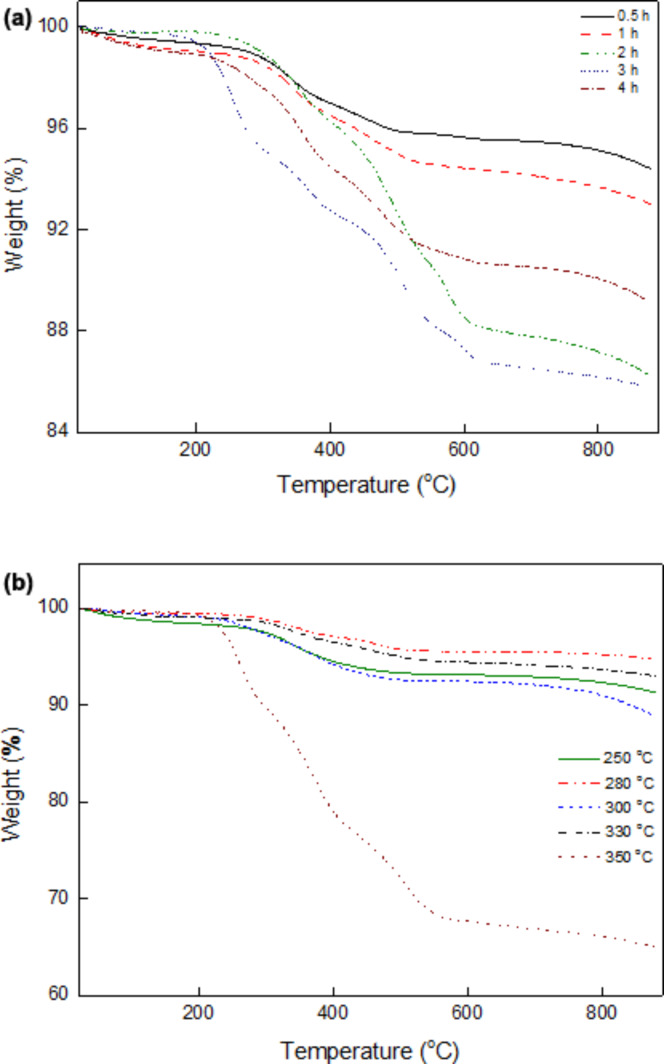
TGA of OM–NaYF_4_:Yb^3+^/Er^3+^ nanoparticles prepared at (a) different reaction temperatures for 1 h and (b) different reaction times at 330 °C.

Another method used to investigate the OM–NaYF_4_:Yb^3+^/Er^3+^ nanoparticles was elemental analysis. The amount of the OM shell on the NaYF_4_:Yb^3+^/Er^3+^ nanoparticles was determined from carbon and nitrogen concentrations according to the following equations: % coating according to C = (% C in sample × 100)/80.74, where 80.74 is % C in OM, and % coating according to N = (% N in sample × 100)/5.23, where 5.23 is % N in OM. The percentage of the OM shell on the nanoparticles prepared at 250–330 °C ranged between approximately 3 and 6 wt % according to both carbon and nitrogen analysis. In contrast, the no. 5 OM–NaYF_4_:Yb^3+^/Er^3+^ nanoparticles obtained at 350 °C (Table 1) contained relatively high amounts of coating (23 and 21 wt % according to C and N analysis, respectively). The OM content on the OM–NaYF_4_:Yb^3+^/Er^3+^ nanoparticles prepared at different reaction temperatures was also determined by TGA (Figure 6a). Small weight losses (ca. 1 wt %) observed upon heating from room temperature to ca. 150 °C were ascribed to evaporation of water and ethanol. Major weight loss was observed at temperatures between 200 and 500 °C due to OM decomposition. If the particles were prepared at 250–330 °C, the amount of OM on the particle surface was rather low (ca. 5–11 wt %). However, the no. 5 particles prepared at 350 °C contained much higher amounts of OM (35 wt %) due to its enhanced adsorption on the surface. Determination of the amount of coating by elemental analysis and TGA was mostly in agreement, but the TGA results were systematically higher, likely due to chemical transformation of inorganic compounds at high temperatures.

**Figure 7:**
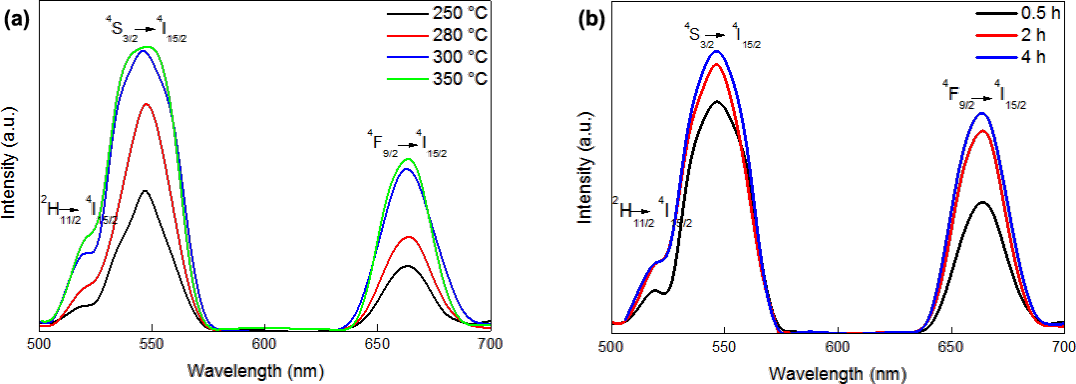
NIR-to-vis upconversion emission spectra of OM–NaYF_4_:Yb^3+^/Er^3+^ nanoparticles excited at 980 nm with a power density of 3 mW/cm^2^. Effect of (a) reaction temperature and (b) time.

**Figure 8:**
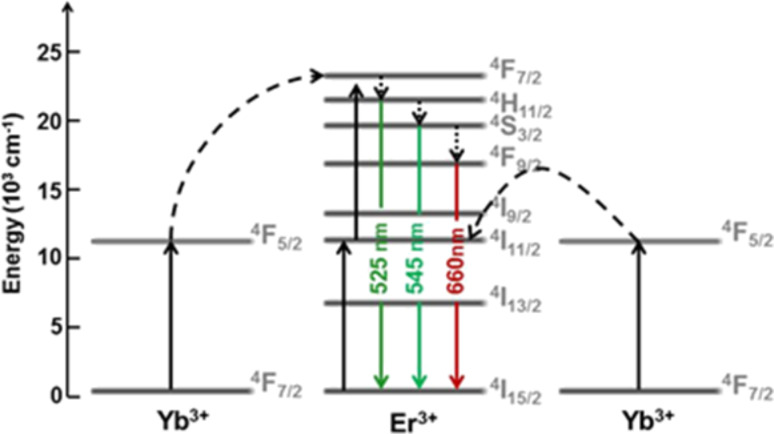
Energy-level diagram of Yb^3+^/Er^3+^ and the upconversion mechanism at 980 nm excitation. The doted and dashed curves represent photon excitation/energy transfer and relaxation, respectively. Only visible emissions are shown [38].

To investigate the optical properties of the OM–NaYF_4_:Yb^3+^/Er^3+^ nanoparticles prepared at different reaction temperatures, the upconversion emission spectra were recorded at 980 nm excitation (Figure 7a). The energy transfer upconversion started after sequential absorption of NIR photons (e.g., 980 nm) by Yb^3+^ ions, leading to the population of Yb^3+^ ions from the ^4^F_7/2_ ground to the ^4^F_5/2_ excited state (Figure 8). This scheme was proposed by Wang, who suggested that the energy was then transferred from the two excited Yb^3+^ ions to the ^4^F_7/2_ state of the neighboring Er^3+^ ion [37]. The ^2^H_11/2_ and ^4^S_3/2_ states of the Er^3+^ ion were populated by nonradiative multiphonon relaxation of the ^4^F_7/2_ state. From these levels, the ion could return directly to the ^4^I_15/2_ ground state or populate the ^4^F_9/2_ state by an additional nonradiative multiphonon relaxation. Consequently, three different Er^3+^ transitions were induced by NIR photons leading to the visible light emission [37]. Hence, the NaYF_4_:Yb^3+^/Er^3+^ nanocrystals exhibited three bands of green (520 and 545 nm) and red (660 nm) upconversion emissions corresponding to ^2^H_11/2_ and ^4^S_3/2_ → ^4^I_15/2_ and ^4^F_9/2_ → ^4^I_15/2_ transitions, respectively. These bands were induced by 4f–4f transitions of the Er^3+^ ions.

Because the red light photons are important for prospective biomedical applications, the *I*_545_/*I*_660_ ratio should be rather low and the total luminescence intensity high. The ratio of green to red emission intensities changed from 2.50 to 1.68. The light emitted by the no. 1 particles prepared at 250 °C had a low intensity due to their cubic crystal structure. However, the no. 2 particles prepared at 280 °C had a higher *I*_545_/*I*_660_ ratio. This can be explained by the different interatomic distances between the Yb^3+^ and Er^3+^ ions in the crystal structure, which changed from cubic to hexagonal. The crystals were not completely hexagonal and contained some cubic impurities, and the distances between Yb^3+^ and Er^3+^ therefore differed, increasing the green light intensity. The red light intensity remained constant due to insufficient energy transfer to Er^3+^ and energy losses between the ions. When the particles were prepared at temperatures above 300 °C, luminescence intensities were slightly higher, indicating that maximum luminescence efficiency was achieved by optimizing the interatomic distance between the absorbers and the emitters.

### Effect of reaction time

In these experiments, OM-NaYF_4_:Yb^3+^/Er^3+^ nanoparticles were prepared at 330 °C and reaction times ranged from 0.5 to 4 h. Other reaction conditions were held constant as described above. The size of the NaYF_4_:Yb^3+^/Er^3+^ nanoparticles of nos. 4 and 6–9 was again approximately 10 nm and the particle size distribution was rather narrow (Table 1 and Table 2). The degree of crystallinity according to XRD (Figure 3b) was approximately 75 wt % (Table 2). A small amorphous halo originated primarily from OM on the nanoparticle surface. In α- and β-phase particles, the presence of an amorphous halo is indicative of a disorder-to-order type cubic-to-hexagonal phase transition [39]. The size of α crystallites ranged from 6 to 6.5 nm, and the size of β-phase crystals ranged between 18–21 nm. Interestingly, diffraction peaks corresponding to the cubic phase were hardly visible in the OM–NaYF_4_:Yb^3+^/Er^3+^ nanoparticles prepared for 3 h, and the size of the crystallites could not be determined. No structural changes were observed by XRD in the particles prepared at different reaction times, indicating that a 4 h reaction time was not sufficient for full conversion of the particles into the hexagonal phase at reaction temperatures below 350 °C. The percentage of coating was again analyzed by both elemental analysis and TGA. The thermal degradation of the OM–NaYF_4_:Yb^3+^/Er^3+^ nanoparticles was similar to that shown in Figure 6a, with the main loss typically occurring between 200 and 600 °C (Figure 6b). According to elemental analysis, the amount of coating ranged from 3 to 7.5 wt %, in accordance with previously described results on the effect of reaction temperature. The content of OM according to TGA was again higher (5.6–14.3 wt %) than when determined by elemental analysis (Table 2). The amount of coating grew as reaction time increased from 0.5 to 3 h.

**Table 2:** Effect of reaction time on OM–NaYF_4_:Yb^3+^/Er^3+^ nanoparticles prepared at 330 °C.

no.	time (h)	*D*_n_ (nm)	PDI	*D*_h_ (nm)	PI	*L*(cubic) (nm)	*L*(hex) (nm)	crystallinity (wt %)	C (wt %)	N (wt %)	coating (wt %)			*I*_545_/*I*_660_
											C	N	TGA	

6	0.5	8	1.20	265	0.54	6.3	19.8	75	2.37	0.11	2.9	2.0	5.6	1.86
4	1	10	1.30	188	0.27	6.4	18.6	74	3.00	0.15	3.7	2.9	7.1	1.79
7	2	11	1.08	200	0.54	6.5	20.8	77	6.12	0.21	7.6	4.0	13.8	1.39
8	3	10	1.15	289	0.69	–	19.9	73	5.88	0.32	7.3	6.1	14.3	1.38
9	4	9	1.09	308	0.63	6.0	17.6	75	5.42	0.31	6.7	5.8	10.9	1.31

*D*_n_: number-average diameter (TEM); PDI: polydispersity index (TEM); *D*_h_: hydrodynamic diameter (DLS); PI: polydispersity (DLS); *L*: average crystallite size (XRD); C: carbon content; N: nitrogen content; TGA: thermogravimetric analysis; *I*_545_/*I*_660_: characteristic upconversion ratio

Visible upconversion spectra of the OM–NaYF_4_:Yb^3+^/Er^3+^ nanoparticles prepared at constant temperature (330 °C) and varying reaction time are shown in Figure 7b. The presence of green and red light was confirmed in three emission spectra of the investigated nanoparticles after two-photon excitation at 980 nm. The intensities of green and red light slightly and significantly increased with increasing reaction time, which can be associated with a highly ordered ion structure of the nanocrystals. The intensity ratio *I*_545_/*I*_660_ decreased with increasing reaction time.

### Modification of the OM–NaYF_4_:Yb^3+^/Er^3+^ nanoparticle surface with SiO_2_

**Figure 9:**
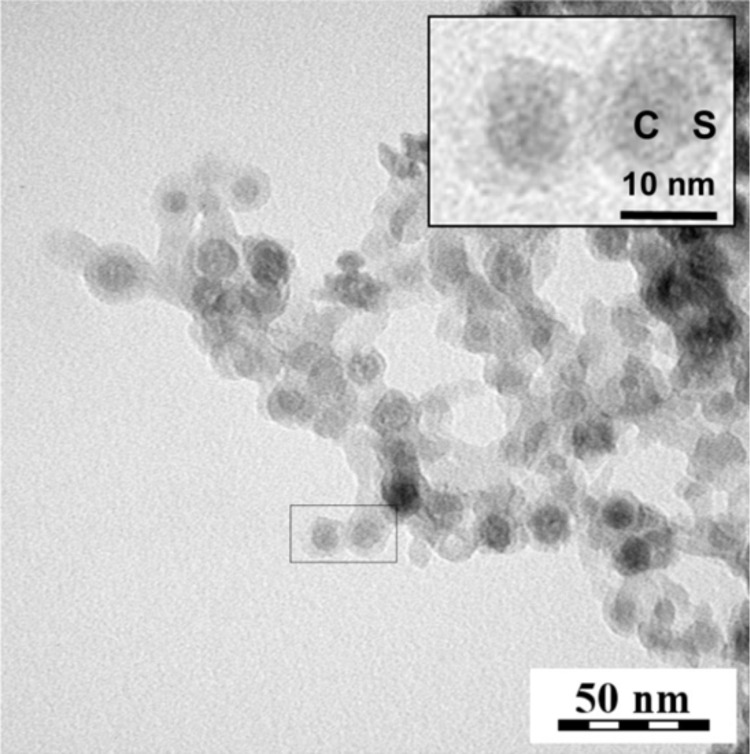
TEM micrograph of NaYF_4_:Yb^3+^/Er^3+^&SiO_2_ nanoparticles. Inset: C – particle core and S – SiO_2_ shell.

**Figure 10:**
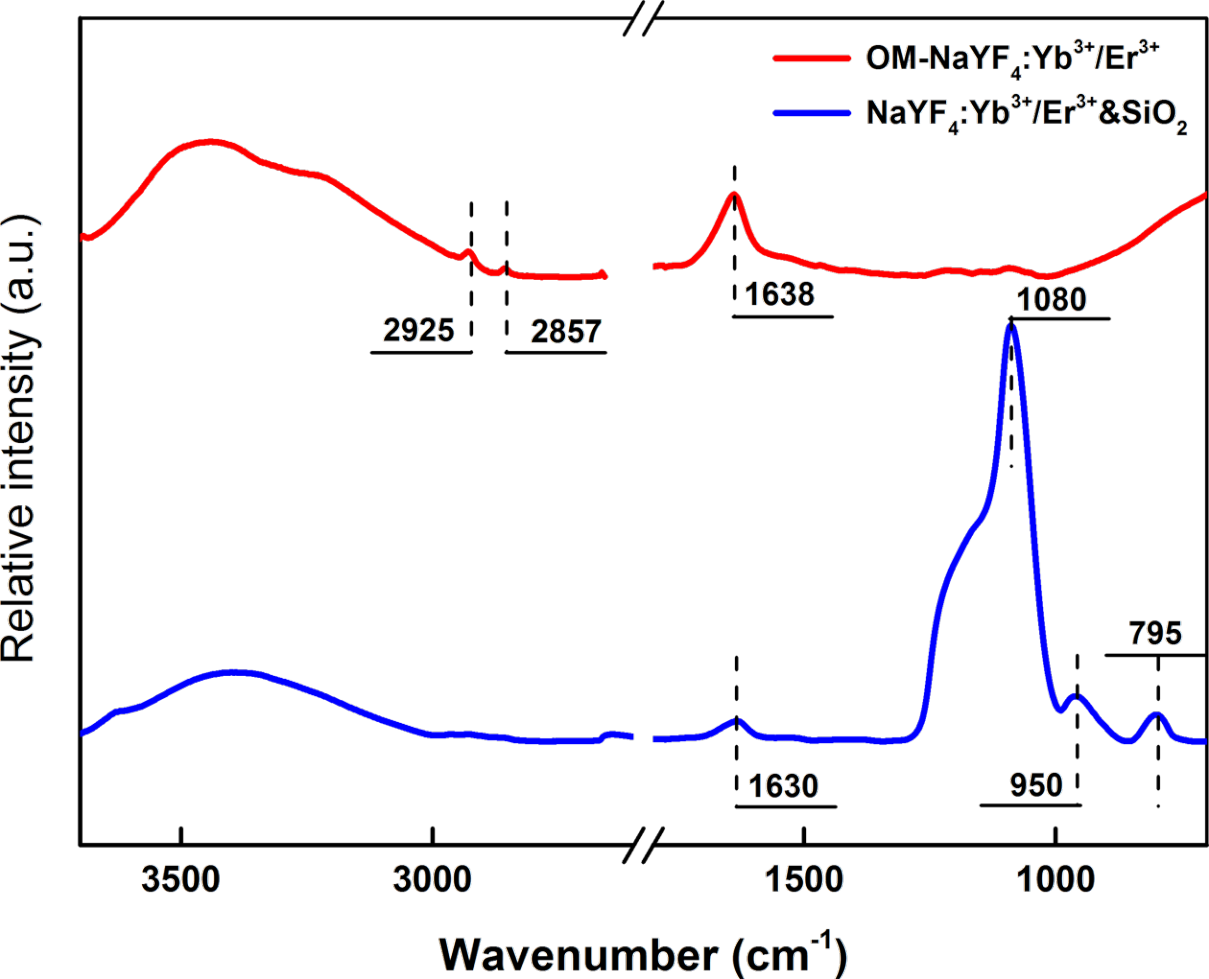
ATR FTIR spectra of OM–NaYF_4_:Yb^3+^/Er^3+^ and NaYF_4_:Yb^3+^/Er^3+^&SiO_2_ nanoparticles.

The OM–NaYF_4_:Yb^3+^/Er^3+^ nanoparticles were easily dispersed in nonpolar organic solvents, such as hexane or toluene, due to the presence of aliphatic OM side chains on the surface. However, the nanoparticles must disperse in aqueous media for biological applications. To disperse the no. 4 NaYF_4_:Yb^3+^/Er^3+^ nanoparticles in water, they were coated with a thin silica shell using a microemulsion technique. TMOS and Igepal CO-520 were used as a precursor and an emulsifier, respectively. Compared with the initial 10 nm OM–NaYF_4_:Yb^3+^/Er^3+^ nanoparticles, the TEM micrograph (Figure 9) showed that the size of the NaYF_4_:Yb^3+^/Er^3+^&SiO_2_ particles had increased to 17 nm due to the presence of the silica shell on the surface. The NaYF_4_:Yb^3+^/Er^3+^&SiO_2_ nanoparticles had a clear core–shell structure with a shell thickness of about 3.5 nm. The presence of the silica shell on the NaYF_4_:Yb^3+^/Er^3+^ nanoparticles was further confirmed by comparing the ATR FTIR spectra of unmodified and modified nanoparticles (Figure 10). The initial OM–NaYF_4_:Yb^3+^/Er^3+^ particles had characteristic absorption bands at 2925 and 2857 cm^−1^ attributed to the asymmetric and symmetric stretching vibrations of the methylene units in the long OM chain [40–41]. After deposition of the silica onto the surface, these peaks were not detected in the ATR FTIR spectrum. New intense absorption bands at 1090 and 795 cm^−1^ in the spectrum of the NaYF_4_:Yb^3+^/Er^3+^&SiO_2_ particles were attributed to the symmetric and asymmetric Si–O–Si stretching vibrations, respectively [42]. An intense 960 cm^−1^ band was also observed in the spectrum of the modified nanoparticles, corresponding to the symmetric stretching vibrations of the Si–OH bonds in the silica [26]. The main evidence for the presence of an OH group was the appearance of intensive broad absorption bands at 3445 and 1630 cm^−1^. These bands are characteristic of OH vibrations. The abovementioned characteristic silica bands verified the presence of SiO_2_ on surface of the NaYF_4_:Yb^3+^/Er^3+^ and successful surface modification.

## Conclusion

OM–NaYF_4_:Yb^3+^/Er^3+^ nanoparticles were synthesized by conventional thermal decomposition of lanthanide trifluoroacetates in OM. Particle morphology was controlled by the careful selection of reaction temperature and time, as evidenced by TEM. XRD and TEM/ED measurements confirmed the presence of cubic α- and hexagonal β-phases in the crystallites. The latter form is preferred in biomedical applications due to its high upconversion efficiency. From a synthetic point of view, temperature had the greatest influence on the resulting crystalline structure. The pure hexagonal phase was obtained at reaction temperatures ≥ 350 °C and annealing times above 1 h. To generate free radicals such as singlet oxygen (destructive to cancer cells) in biological experiments, the nanoparticles should emit photons at 660 nm to excite phthalocyanine, a typical photosensitizer [43]. Upconversion OM–NaYF_4_:Yb^3+^/Er^3+^ nanoparticles were excited by near-infrared light at 980 nm, i.e., at the Yb^3+^ absorption maximum. Photons were emitted at 520, 545 and 660 nm in the fluorescence spectra of the OM–NaYF_4_:Yb^3+^/Er^3+^ nanoparticles. The NaYF_4_:Yb^3+^/Er^3+^ nanoparticles were successfully coated with a silica shell using the reverse microemulsion method, making them dispersible in water and promising candidates for applications in biology and medicine.
